# Supplementary material for: Pause characteristics of sentence production in Parkinson’s disease: Insights from sentence complexity and length
Source: PLoS One. 2026 Apr 23;21(4):e0330808. doi: 10.1371/journal.pone.0330808 (PMC13105342; doi:10.1371/journal.pone.0330808)
Supplement: S1 File — Appendix 1: Criteria for Complexity of Sentence in Rainbow Passage. Based on Dede and Salis (2020). Appendix 2: Categorisation of Sentences in Rainbow Passage. (DOCX) [file pone.0330808.s001.docx]

# **Appendices**

**Appendix 1: Criteria for Complexity of Sentence in Rainbow Passage.** Based on Dede and Salis (2020)

| Simple | Complex |
| --- | --- |
| One independent clause containing a subject and a predicate | One or more dependent clauses to an independent clause |
| May contain compound nouns and verbs | Include at least one embedded clause |
| Can contain coordinating conjunctions (and, but, or) | Either have a subordinating clause (because, when, although) or a relative pronoun (that, which who etc.) |

Length of Sentence was determined by using the mean of the sentence length, with Long being above 17.21 words a sentence and short sentences below 17.21 words per sentence

i.e. 327 words/19 sentences = 17.21 words per sentence

## **Appendix 2: Categorisation of Sentences in Rainbow Passage**

Key: C = Complex, S = Simple

**The Rainbow Passage**

C - When the sunlight strikes raindrops in the air, they act like a prism and form a rainbow.

S - The rainbow is a division of white light into many beautiful colours.

S - These take the shape of a long, round arch, with its path high above and its two ends apparently beyond the horizon.

C - There is, according to legend, a boiling pot of gold at one end.

S - People look, but no one ever finds it.

C - When a man looks for something beyond his reach, his friends say he is looking for the pot of gold at the end of the rainbow.

S - Throughout the centuries people have explained the rainbow in various ways.

S - Some have accepted it as a miracle without physical explanation.

C - To the Hebrews it was a token that there would be no more universal floods.

C - The Greeks used to imagine that it was a sign from the gods to foretell war or heavy rain.

C - The Norsemen considered the rainbow as a bridge over which the gods passed from earth to their home in the sky.

S – Others have tried to explain the phenomenon physically.

C - Aristotle thought that the rainbow was caused by reflection of the sun's rays by the rain.

C - Since then, physicists have found that it is not reflection, but refraction by the raindrops, which causes the rainbow.

S - Many complicated ideas about the rainbow have been formed.

C - The difference in the rainbow depends considerably upon the size of the drops, where the width of the coloured band increases as the size of the drops increases.

S - The actual primary rainbow observed is said to the effect of superimposition of a number of bows.

C - If the red of the second bow falls upon the green of the first, the result is to give a bow with abnormally wide yellow band, since red and green light when mixed form yellow.

C - This is a very common type of bow, one showing mainly red and yellow, with little or no green or blue.

**Key:** S = short, L = Long

**The Rainbow Passage**

S - When the sunlight strikes raindrops in the air, they act like a prism and form a rainbow.

S - The rainbow is a division of white light into many beautiful colours.

L - These take the shape of a long, round arch, with its path high above and its two ends apparently beyond the horizon.

S - There is, according to legend, a boiling pot of gold at one end.

S - People look, but no one ever finds it.

L - When a man looks for something beyond his reach, his friends say he is looking for the pot of gold at the end of the rainbow.

S - Throughout the centuries people have explained the rainbow in various ways.

S - Some have accepted it as a miracle without physical explanation.

S - To the Hebrews it was a token that there would be no more universal floods.

L - The Greeks used to imagine that it was a sign from the gods to foretell war or heavy rain.

L - The Norsemen considered the rainbow as a bridge over which the gods passed from earth to their home in the sky.

S – Others have tried to explain the phenomenon physically.

S - Aristotle thought that the rainbow was caused by reflection of the sun's rays by the rain.

L - Since then, physicists have found that it is not reflection, but refraction by the raindrops, which causes the rainbows.

S - Many complicated ideas about the rainbow have been formed.

L - The difference in the rainbow depends considerably upon the size of the drops, where the width of the coloured band increases as the size of the drops increases.

S - The actual primary rainbow observed is said to the effect of superimposition of a number of bows.

L - If the red of the second bow falls upon the green of the first, the result is to give a bow with abnormally wide yellow band, since red and green light when mixed form yellow.

L - This is a very common type of bow, one showing mainly red and yellow, with little or no green or blue.
