# Supplementary material for: Pause characteristics of sentence production in Parkinson’s disease: Insights from sentence complexity and length
Source: PLoS One. 2026 Apr 23;21(4):e0330808. doi: 10.1371/journal.pone.0330808 (PMC13105342; doi:10.1371/journal.pone.0330808)
Supplement: S1 Table — (DOCX) [file pone.0330808.s002.docx]

Supplementary Material 1: Demographic information of Parkinson group

| PD | Post 16 Education (years) | Time Post PD Diagnosis (months) | MoCA Score | Dysarthria Severity Rating |
| --- | --- | --- | --- | --- |
| PD01 | 5 | 24 | 28 | 57 |
| PD02 | 5 | 148 | 30 | 63 |
| PD03 | 5 | 183 | 27 | 50 |
| PD04 | 6 | 26 | 28 | 52 |
| PD05 | 5 | 87 | 24 | 76 |
| PD06 | 6 | 41 | 30 | 45 |
| PD07 | 1 | 14 | 22 | 58 |
| PD08 | 5 | 211 | 23 | 58 |
| PD09 | 5 | 123 | 29 | 60 |
| PD10 | 2 | 39 | 29 | 51 |
| PD11 | 5 | 194 | 25 | 66 |
| PD12 | 6 | 71 | 28 | 38 |
| PD13 | 6 | 82 | 30 | 63 |
| PD14 | 5 | 231 | 29 | 54 |
| PD15 | 5 | 39 | 25 | 37 |
| PD16 | 5 | 27 | 24 | 55 |
| PD17 | 5 | 63 | 22 | 95 |
| PD18 | 5 | 51 | 23 | 53 |
| PD19 | 6 | 3 | 27 | 39 |
| PD20 | 2 | 23 | 28 | 72 |
| PD21 | 2 | 40 | 30 | 41 |
| PD22 | 4 | 53 | 28 | 58 |
| PD23 | 6 | 21 | 23 | 45 |
| PD24 | 1 | 22 | 27 | 85 |
| PD25 | 6 | 10 | 27 | 37 |
| PD26 | 9 | 47 | 26 | 57 |
| PD27 | 0 | 24 | 27 | 89 |
| PD28 | 6 | 49 | 26 | 58 |
| PD29 | 2 | 108 | 28 | 44 |
| PD30 | 9 | 129 | 27 | 46 |
| PD31 | 3 | 78 | 28 | 40 |
| PD32 | 8 | 88 | 26 | 69 |
| Mean | 4.72 | 73.41 | 26.69 | 55 |
| Standard Deviation | 2.16 | 62.1441 | 2.40 | 13.8 |
