# Supplementary material for: Pause characteristics of sentence production in Parkinson’s disease: Insights from sentence complexity and length
Source: PLoS One. 2026 Apr 23;21(4):e0330808. doi: 10.1371/journal.pone.0330808 (PMC13105342; doi:10.1371/journal.pone.0330808)
Supplement: S2 Table — (DOCX) [file pone.0330808.s003.docx]

## Supplementary Material 2: Demographic information of Control group

| Control | MoCA score | Post-16 Education (years) |
| --- | --- | --- |
| OC01 | 30 | 6 |
| OC02 | 26 | 5 |
| OC03 | 27 | 5 |
| OC04 | 25 | 8 |
| OC05 | 26 | 6 |
| OC06 | 28 | 2 |
| OC07 | 26 | 2 |
| OC08 | 26 | 0 |
| OC09 | 29 | 6 |
| OC10 | 30 | 6 |
| OC11 | 26 | 5 |
| OC12 | 29 | 6 |
| OC13 | 29 | 2 |
| OC14 | 29 | 3 |
| OC15 | 29 | 5 |
| OC16 | 29 | 5 |
| OC17 | 30 | 5 |
| OC18 | 30 | 2 |
| OC19 | 30 | 3 |
| OC20 | 29 | 0 |
| OC21 | 28 | 5 |
| OC22 | 30 | 5 |
| OC23 | 29 | 5 |
| OC24 | 29 | 1 |
| OC25 | 30 | 5 |
| OC26 | 30 | 5 |
| OC27 | 28 | 5 |
| OC28 | 29 | 2 |
| OC29 | 29 | 0 |
| OC30 | 28 | 0 |
| OC31 | 30 | 0 |
| OC32 | 29 | 0 |
| OC33 | 27 | 6 |
| OC34 | 29 | 5 |
| OC35 | 29 | 8 |
| OC36 | 28 | 5 |
| OC37 | 27 | 7 |
| OC38 | 26 | 0 |
| OC39 | 28 | 2 |
| Mean | 28.36 | 3.79 |
| Standard Deviation | 1.46 | 2.43 |
