# Supplementary material for: Pause characteristics of sentence production in Parkinson’s disease: Insights from sentence complexity and length
Source: PLoS One. 2026 Apr 23;21(4):e0330808. doi: 10.1371/journal.pone.0330808 (PMC13105342; doi:10.1371/journal.pone.0330808)
Supplement: S3 Table — based on DeDe & Salis (2019) and Reed (2020) – Only showing codes used in from initial criteria. (DOCX) [file pone.0330808.s004.docx]

Supplementary Material 3: Praat Coding Criteria

| Classification | Definition |
| --- | --- |
| Silent Pauses (SP) | Absence of acoustic signal for a period greater than or equal to 200ms |
| Filled Pauses (FP) | Phoneme vocalisations such as “uh”, “er”, “eh”, “uhm”, and “mm” |
| Mazes (Mz) | Verbal segments that was used in non-propositional function (e.g., “something of other”, “I’m not sure”, “I don’t know”, “like”, “ok”, “oh god”).  If the maze was accompanied by other phrases in a clearly identified linguistic structure and conveyed propositional meaning, then the segments was not coded for a maze. For example, in the sentences “I’m not sure how many sisters there were” or “I don’t know what happened next”, the phrases “I’m not sure”, I don’t know” were not counted as mazes. However, if these phrases were uttered on their own, then they would be classified as mazes. Unintelligible segments were also logged as mazes but were excluded from word count. |
| Repetitions (RP) | Identical words or phrases that occurred sequentially two or more times. For example, “the, the” is one repetition whereas “the, the, the” are two repetitions. Repetitions could be separated by a silent or a filled paus |
| Revisions (Rv) | Words or phrases that had a propositional function and it was that the person revised a previously produced word or phrase. For example, in the phrase “the, the, this house”, “this” is a revision. Revisions could be separated by a silent or filled pause for example in the phrases, “got all of [silent pause] cleaned the floor and stuff” the segment “cleaned the floor and stuff” is a revision. |

Note 1: Based on DeDe & Salis (2019) and Reed (2020) - Only showing codes used in from initial criteria.
