# Supplementary material for: Pause characteristics of sentence production in Parkinson’s disease: Insights from sentence complexity and length
Source: PLoS One. 2026 Apr 23;21(4):e0330808. doi: 10.1371/journal.pone.0330808 (PMC13105342; doi:10.1371/journal.pone.0330808)
Supplement: S4 Table — (DOCX) [file pone.0330808.s005.docx]

## Supplementary Material 4: Normality Test Results

### OC Group

| Sentence type Statistic | Kolmogorov-Smirnov | | | Shapiro-Wilk Test | | |
| --- | --- | --- | --- | --- | --- | --- |
|  | Statistic | df | Sig. | Statistic | df | Sig. |
| Short mean number | .077 | 38 | .200* | .978 | 38 | .650 |
| Short mean duration | .128 | 38 | .119 | .966 | 38 | .298 |
| Long mean number | .103 | 38 | .200* | .975 | 38 | .530 |
| Long mean duration | .091 | 38 | .200* | .976 | 38 | .575 |
| Simple mean number | .137 | 38 | .068 | .949 | 38 | .085 |
| Simple mean duration | .064 | 38 | .200* | .986 | 38 | .903 |
| Complex mean number | .102 | 38 | .200* | .971 | 38 | .431 |
| Complex mean duration | .118 | 38 | .200* | .954 | 38 | .120 |
| Between sentence mean duration | .077 | 38 | .200* | .987 | 38 | .939 |
| Total number pauses | .095 | 38 | .200* | .976 | 38 | .583 |

*. This is a lower bound of the true significance

### PD Group

| Sentence type Statistic | Kolmogorov-Smirnov | | | Shapiro-Wilk Test | | |
| --- | --- | --- | --- | --- | --- | --- |
|  | Statistic | df | Sig. | Statistic | df | Sig. |
| Short mean number | .193 | 30 | .006 | .939 | 30 | .087 |
| Short mean duration | .174 | 30 | .021 | .907 | 30 | .012 |
| Long mean number | .225 | 30 | <.001 | .843 | 30 | <.001 |
| Long mean duration | .138 | 30 | .149 | .854 | 30 | <.001 |
| Simple mean number | .169 | 30 | .018 | .920 | 30 | .027 |
| Simple mean duration | .169 | 30 | .029 | .929 | 30 | .047 |
| Complex mean number | .178 | 30 | .017 | .879 | 30 | .003 |
| Complex mean duration | .167 | 30 | .033 | .871 | 30 | .002 |
| Between sentence mean duration | .115 | 30 | .200* | .966 | 30 | .444 |
| Total number pauses | .239 | 30 | <.011 | .885 | 30 | .004 |
