# Supplementary material for: Pause characteristics of sentence production in Parkinson’s disease: Insights from sentence complexity and length
Source: PLoS One. 2026 Apr 23;21(4):e0330808. doi: 10.1371/journal.pone.0330808 (PMC13105342; doi:10.1371/journal.pone.0330808)
Supplement: Attachment — Submitted filename: Review PLOS One pausing 2025.docx [file pone.0330808.s006.docx]

This manuscript focused on pausing behaviors in people with Parkinson’s disease. In general, this is an interesting topic and relevant to improving the development and understanding of speech-based biomarkers in people with PD. The concept behind the study is strong, and it could add to the several papers that have been published about the mechanisms and clinical implications of pausing during speech in PD. However, the authors do not adequately address existing literature and the synthesis and interpretation of their findings is weakened as a result. There are also some inconsistencies and areas of decreased clarity in the methodology.

Introduction:

The authors touch on several unmet needs in understanding and treating speech dysfunction in people with PD, but the topics are somewhat tangential. The connection between speech-based markers and impact on clinical practice could be made more direct and clearer. The authors could make a stronger case for why pause characterization may improve evaluation and treatment. There are many studies about pause timing in PD, so the statement that less attention has been paid is not well supported (see several Huber et al. papers, Smith et al. 2018, Andrade et al. 2023 among others).

Lines 83-87 would be strengthened by reviewing this literature and explaining the current state of knowledge about speech rate, articulation rate, and pause behaviors with more up-to-date literature. It may be helpful to define these terms and how they relate to pause measures and their implementation in research paradigms.

Methods:

Later parts of the paper discuss syllable content (lines 426-432) but this is not mentioned in the methods as a criteria used to categorize the sentence types. Please clarify.

The authors should state their rationale for pause duration thresholds.

Importantly, simple/complex and short/long sentence categorizations are not mutually exclusive. This fact makes the findings more difficult to interpret.

Please state how many raters there were.

Discussion:

There are missing topic areas that would add to the synthesis of findings and place their work in the greater context of the literature. One is respiratory pauses and another is grammatical pauses. The paper would be stronger with a more well-conceived hypothesis to explain their findings in light of these different pause behaviors and their mechanisms. Finally, speech task type is not addressed at all. Mention of prior studies should specify if the speech task was reading or spontaneous speech, because studies done using spontaneous speech would understandably demonstrate inconsistencies compared to this paper using reading only. Notably, lexical retrieval and word selection (line 436) do not apply to read speech.

There is no discussion of any detected patterns concerning filled and unfilled pauses, but this would make the findings more interesting and help explain the behavioral driving factors of these pauses in the PD group.

In general, in a few places the authors note “disease severity” when “dysarthria severity” is really what can be measured under their methodology.

Additional limitations include the small number of females in the sample, and wide disease duration range, and the use of MoCA as cognitive categorization practice. Importantly, there are no motor symptom measures and there seem to be differences between the data collection procedures at the two sites.

The pause duration threshold, which was relatively short compared to the approach used in prior studies, should be discussed. How might the inclusion of very short pauses have impacted the results?

In lines 439-440, this sentence has several issues. First, it is not clear why pause duration would be affected by articulatory movements as the articulators are not active during silent intervals. Second, speech rate and articulation timing have been shown to be related to cognitive function in people with PD in several studies.
