# Supplementary material for: Pause characteristics of sentence production in Parkinson’s disease: Insights from sentence complexity and length
Source: PLoS One. 2026 Apr 23;21(4):e0330808. doi: 10.1371/journal.pone.0330808 (PMC13105342; doi:10.1371/journal.pone.0330808)
Supplement: Attachment — Submitted filename: Response to Reviewers.docx [file pone.0330808.s008.docx]

**Manuscript ID:** PONE-D-25-42565

Responses to the reviewers’ comments

We thank the two reviewers and the Editors of PLOS ONE for the time and effort devoted to evaluating our manuscript. We are grateful for the constructive and insightful comments, which have been carefully considered in revising our work. We appreciate the opportunity to resubmit our manuscript. In response to the reviewers’ suggestions, we have incorporated the requested additional information, provided detailed point-by-point responses to all comments, and thoroughly revised the manuscript. Major changes have been clearly highlighted in the revised version in red. We remain fully committed to addressing any further concerns raised by the reviewers or editors and to correcting any inadvertent errors. Below, we restate each comment followed by our detailed response.

**Reviewer #1:** See attached. Re: item 1 above, the paper is modestly technically sound. The authors need to address questions about the methodology and identify areas of limited rigor such as small sample of female participants.

**Response:** Thank you for this observation. We acknowledge that the number of female participants was smaller than that of male participants. Recruiting individuals with Parkinson’s disease who are able to remain off medication for 12 hours is particularly challenging; therefore, we did not further restrict enrollment based on sex. Additionally, Parkinson’s disease is more commonly diagnosed in males than females, with an approximate ratio of 2:1 (Van Den Eeden et al., 2003). However, it remains unclear whether this disparity reflects true differences in disease prevalence or sex-related differences in symptom presentation and health-seeking behavior, which may lead to underdiagnosis in females. Addressing these possibilities would require further investigation beyond the scope of the present study (Miller & Cronin‐Golomb., 2010).

Attachment:

This manuscript focused on pausing behaviors in people with Parkinson’s disease. In general, this is an interesting topic and relevant to improving the development and understanding of speech-based biomarkers in people with PD. The concept behind the study is strong, and it could add to the several papers that have been published about the mechanisms and clinical implications of pausing during speech in PD. However, the authors do not adequately address existing literature and the synthesis and interpretation of their findings is weakened as a result. There are also some inconsistencies and areas of decreased clarity in the methodology.

Introduction:

The authors touch on several unmet needs in understanding and treating speech dysfunction in people with PD, but the topics are somewhat tangential. The connection between speech-based markers and impact on clinical practice could be made more direct and clearer. The authors could make a stronger case for why pause characterization may improve evaluation and treatment. There are many studies about pause timing in PD, so the statement that less attention has been paid is not well supported (see several Huber et al. papers, Smith et al. 2018, Andrade et al. 2023 among others).

Lines 83-87 would be strengthened by reviewing this literature and explaining the current state of knowledge about speech rate, articulation rate, and pause behaviors with more up-to-date literature. It may be helpful to define these terms and how they relate to pause measures and their implementation in research paradigms.

**Response:** We thank the reviewer for bringing this point. We have now revised the Introduction to include a more direct link between the pause characteristics and the evaluation treatment of speech in PD. Please see below and also in the manuscript lines 80-99:

“In PD, **speech timing** is commonly disrupted, with **pause behaviour** emerging as a more consistent feature than changes in speech or articulation rate. **Speech rate** refers to the overall speed of speech production, typically measured as syllables or words per second **including pauses**, whereas **articulation rate** reflects the speed of articulatory movements **excluding pauses**, and **pause timing** encompasses the frequency, duration, and placement of silent intervals during speech. Speech rate is often reduced in PD, although findings are mixed and influenced by task type and disease stage, while articulation rate appears relatively preserved, suggesting that articulatory execution is less affected than higher-level planning and timing processes (Blanchet & Snyder, 2009; Illner et al., 2022; Moreau & Pinto, 2019). In contrast, pause duration and distribution constitute robust markers of PD speech, reflecting dysfunction within basal ganglia–cortical circuits involved in speech sequencing and timing, as supported by evidence that deep brain stimulation modulates pause characteristics (Ahn et al., 2014; Guenther, 2016). Abnormal pausing may also reflect linguistic and cognitive difficulties, including lexical retrieval, syntactic processing, and executive control, and has been linked to cognitive function in PD, highlighting its potential value as a speech-based marker of cognitive decline (Bohsali & Crosson, 2016; Lee et al., 2019; Andrade et al., 2023). Clinically, these findings suggest that assessment and management of speech in PD should move beyond global speech rate measures to include detailed analysis of pause timing, particularly in connected speech, and that interventions focused solely on slowing speech may be less effective than approaches targeting planning, prosodic phrasing, and respiratory–speech coordination (Knowles et al., 2021; Lowit et al., 2018; for a review, see Fumel et al., 2024).”

Methods:

Later parts of the paper discuss syllable content (lines 426-432) but this is not mentioned in the methods as a criteria used to categorize the sentence types. Please clarify.

**Response:** This paragraph describes the variables under investigation, namely sentence complexity (simple vs. complex) and sentence length (short vs. long; see Appendix 2 for the classification criteria). The number of syllables is used as an index of sentence length, with longer sentences containing a higher syllable count. To improve clarity, we have rephrased this paragraph accordingly. As noted at the end of the paragraph, syllable count was not explicitly controlled for in the present study and may therefore represent a potential confound that warrants further investigation please refer to lines 442-451.

The authors should state their rationale for pause duration thresholds.

**Response:** As we pointed in the manuscript pauses were set at a boundary of 200 ms, based on recommendations by DeDe and Salis (2020) to capture shorter pauses while excluding those that likely corresponded to breath or articulation. We have expanded here to include the rationale for pause duration threshold clear. Please see below and refer to lines 208-224:

“A TextGrid was created in Praat to mark silent pauses, defined as intervals exceeding 150 ms based on (Alvar et al., 2019). For this study, pauses were set at a boundary of 200 ms, based on recommendations by DeDe and Salis (2020), to capture shorter pauses while excluding those that likely corresponded to breath or articulation. A **200-ms pause duration threshold** is commonly justified in studies of PD speech because it provides a principled boundary between **brief articulatory gaps** and **functionally meaningful pauses** related to speech planning, timing, and cognitive–linguistic processing (Lee et al., 2019). Silent intervals shorter than approximately 200 ms are generally considered part of normal articulatory coordination and segmental timing, whereas pauses exceeding this duration are more likely to be perceived by listeners as disruptions to speech flow and to reflect higher-level processes such as lexical retrieval, syntactic planning, or respiratory control (Goldman-Eisler, 1968; Campione & Véronis, 2002). In PD, basal ganglia dysfunction is known to affect speech timing and sequencing, making pauses above this threshold particularly relevant as markers of impaired motor planning and cognitive–linguistic integration (Guenther, 2016; Ahn et al., 2014).Using a 200-ms cutoff therefore helps isolate pauses that are sensitive to PD-related speech impairments while excluding micro-silences that are unlikely to be clinically or theoretically meaningful, and this threshold has been widely adopted in analyses of connected speech in PD and related populations (Campione & Véronis, 2002; Lee et al., 2019).”

Importantly, simple/complex and short/long sentence categorizations are not mutually exclusive. This fact makes the findings more difficult to interpret.

## **Response:** Thanks for this point. We agree that considering the complexity and length of sentences makes the findings more difficult to interpret, and that’s why we have compared the duration and the number of pauses across sentence complexity (simple vs. complex) and sentence length (short vs. long) as two factors and interpreted our results for each one separately as well (Research Question 2: Does sentence complexity and length affect pause behaviour?)

Please state how many raters there were.

**Response:** This now has been added to the manuscript, see line 241:

“Inter-rater reliability was assessed using qualitative ratings for the number of pauses and duration of pauses by two independent raters.”

Discussion:

There are missing topic areas that would add to the synthesis of findings and place their work in the greater context of the literature. One is respiratory pauses and another is grammatical pauses. The paper would be stronger with a more well-conceived hypothesis to explain their findings in light of these different pause behaviors and their mechanisms. Finally, speech task type is not addressed at all. Mention of prior studies should specify if the speech task was reading or spontaneous speech, because studies done using spontaneous speech would understandably demonstrate inconsistencies compared to this paper using reading only. Notably, lexical retrieval and word selection (line 436) do not apply to read speech.

**Response:** Thanks for these thoughtful points that will undoubtedly improve the Discussion and position it in the greater context. We have now revised the Discussion to include the respiratory pauses and grammatical pauses. In addition, we have included on the speech task type comparing reading to other types of tasks that previous studies have used. We have also removed this sentence on line 436 from the revised version of the manuscript: “These results align with expectations, suggesting that cognitive ability influences the efficiency of lexical retrieval and word selection in PD speech”. Please refer to lines 455-477 in the revised version of the manuscript and see below:

“Previous research has shown that individuals with PD exhibit longer and more frequent silent pauses, reduced use of filled pauses, and poorer alignment of respiratory pauses with grammatical boundaries, reflecting disruptions in linguistic planning, automatic cueing, and respiratory–speech coordination (Rosen et al., 2010; Huber & Darling-White, 2017; Lowit et al., 2018). Much of this evidence comes from spontaneous or semi-spontaneous speech tasks, such as narratives or picture descriptions, which place high demands on lexical retrieval, discourse planning, and internal timing, thereby amplifying pause abnormalities in PD (Rosen et al., 2010; Ash et al., 2012). Silent pauses in PD have been associated with increased cognitive–linguistic load and lexical access difficulty, whereas filled pauses—typically serving as planning buffers—are reduced, likely due to basal ganglia dysfunction affecting internally generated cues (Rosen et al., 2010; Ash et al., 2012). This study extends this literature by examining pausing during a controlled reading task (Rainbow Passage), in which lexical content and syntactic structure are externally provided, reducing discourse-level planning demands while preserving sentence-level formulation and respiratory coordination requirements. Despite these reduced demands, sentence complexity (simple vs. complex) and sentence length (short vs. long) systematically modulated pause behavior in PD, with longer and more complex sentences eliciting longer silent pauses, particularly within sentences. Although respiratory pauses were not directly measured, the altered distribution of silent pauses within grammatical structures is consistent with prior findings of impaired coordination between respiration and linguistic planning in PD (Lowit et al., 2018). Together, these findings indicate that pause abnormalities in PD are evident across speech task types and reflect an interaction between motor–respiratory constraints and higher-level linguistic planning, with sentence complexity and length exacerbating these effects even during passage reading.”

There is no discussion of any detected patterns concerning filled and unfilled pauses, but this would make the findings more interesting and help explain the behavioral driving factors of these pauses in the PD group.

**Response:** We agree with this point and have added the discussion on filled and silent/unfilled pauses with the pervious point that you brought up. Please see the response above and refer to the revised version of the manuscript lines 455-477.

In general, in a few places the authors note “disease severity” when “dysarthria severity” is really what can be measured under their methodology.

**Response:** Thank you for this comment. We agree that our measurements reflect **dysarthria severity** rather than overall disease severity. We have revised the manuscript accordingly and replaced all instances of “disease severity” with “dysarthria severity.”

Additional limitations include the small number of females in the sample, and wide disease duration range, and the use of MoCA as cognitive categorization practice. Importantly, there are no motor symptom measures and there seem to be differences between the data collection procedures at the two sites.

**Response:** Thanks for these thoughtful comments. We acknowledge that the number of female participants in the present study was smaller than the number of male participants. Recruitment of individuals with Parkinson’s disease (PD) who are able to remain off dopaminergic medication for 12 hours is particularly challenging and tends to limit enrollment to individuals with mild to moderate disease severity. For this reason, we did not further restrict participation based on sex or disease duration. In addition, PD is more frequently diagnosed in males than in females, with an approximate male-to-female ratio of 2:1 (Van Den Eeden et al., 2003). However, it remains unclear whether this disparity reflects true differences in disease prevalence or sex-related differences in symptom presentation and health-seeking behaviors that may contribute to underdiagnosis in females, an issue that warrants further investigation beyond the scope of the present study (Miller et al., 2010).

To reduce potential confounds related to cognition, we controlled for the range of MoCA scores within the PD group, indicating that participants did not exhibit severe cognitive impairment that could influence speech performance beyond PD-related effects. We did not collect standardized measures of overall motor severity (e.g., UPDRS or Hoehn and Yahr staging); however, given that participants were able to tolerate a 12-hour medication withdrawal, we infer that the sample primarily consisted of individuals with mild to moderate motor symptom severity. We acknowledge the absence of formal motor severity measures as a limitation and note that future studies would benefit from incorporating comprehensive assessments of motor symptoms beyond speech.

With respect to data collection procedures, we acknowledge that data were collected in different formats (in person at McGill University and online at the University of Reading due to COVID-19 restrictions). Nevertheless, we took care to ensure that identical protocols were followed across sites. These methodological considerations and limitations have now been explicitly acknowledged in the Limitations and future directions section of the manuscript (lines 518-544) and below:

“PD group included fewer female than male participants. Although this imbalance is consistent with epidemiological reports indicating a higher diagnosis rate in males (Van Den Eeden et al., 2003), it may also reflect sex-related differences in symptom presentation or health-seeking behaviors that contribute to underdiagnosis in females (Miller & Cronin‐Golomb., 2010). Recruitment was further constrained by the requirement that participants remain off dopaminergic medication for 12 hours, which limited enrollment primarily to individuals with mild to moderate disease severity and precluded additional stratification by sex or disease duration. Future studies with larger and more diverse samples are needed to better examine potential sex-related effects in PD speech and pausing behavior.

Additionally, although cognitive status was partially controlled by restricting the range of MoCA scores in the PD group, comprehensive neuropsychological assessments were not conducted. As cognitive-linguistic factors can influence speech production and pausing patterns, future work would benefit from more detailed cognitive profiling to better dissociate speech motor impairments from broader cognitive contributions. In addition, standardized measures of overall motor severity, such as the Movement Disorders Society - **Unified Parkinson’s Disease Rating Scale (MDS-UPDRS;** Goetz, 2009) and the **Hoehn and Yahr staging system** (Hoehn & Yahr, 1967), were not collected. While participants’ ability to tolerate a 12-hour medication withdrawal suggests predominantly mild to moderate motor symptom severity, the absence of formal motor ratings limits the ability to directly relate pausing behavior to global motor impairment. Future studies should incorporate comprehensive motor assessments alongside speech measures.

Moreover, data were collected using different formats across sites, with in-person recordings at McGill University and online data collection at the University of Reading due to COVID-19 restrictions. Although identical experimental protocols were followed to the extent possible, differences in recording environments may have introduced variability. Future research would benefit from fully standardized data collection methods or from explicitly modeling recording modality as a factor in the analysis.”

The pause duration threshold, which was relatively short compared to the approach used in prior studies, should be discussed. How might the inclusion of very short pauses have impacted the results?

**Response:** We thank the reviewer for this important point. In our study, the pause duration threshold was set relatively short compared to some prior work in order to capture **both brief hesitations and longer, linguistically meaningful pauses**. Including very short pauses allows for a more complete characterization of the temporal structure of speech and can be particularly informative in PD, where subtle disruptions in speech motor control may manifest as brief interruptions that would be missed with a higher threshold. We acknowledge that using a shorter threshold likely increased the total number of detected pauses and may have slightly reduced the average pause duration compared to studies using longer thresholds. However, the primary comparisons in our study—effects of sentence complexity and sentence length—remain valid, as these manipulations affect **relative pause duration and frequency across conditions**, and all pauses were detected consistently across participants. We have added a discussion of this issue to the manuscript (lines 488-503) to clarify the rationale for our threshold and its potential impact on the results. see below:

“In the present study, we used a relatively short pause duration threshold to capture a broader range of speech interruptions, including brief hesitations that may reflect subtle motor or planning disruptions in PD. Prior research on pausing in speech has varied widely in how pauses are defined. For example, studies of spontaneous speech have commonly used thresholds around **100–250 ms** to distinguish short articulatory or motor pauses from longer cognitive–linguistic pauses, with 250 ms often taken as a benchmark for long pauses reflecting planning processes (Lee et al., 2019; DeDe et al., 2025; DeDe & Salis, 2020), and some work in PD has used even shorter thresholds (e.g., ~15 ms) to enable fine‑grained analysis of hesitation patterns (Whitfield & Gravelin, 2019; Rosen et al., 2010). Including very short pauses likely increased the number of detected pauses and lowered mean pause durations compared to studies using longer cut‑offs. However, because the same threshold was applied consistently across all sentences and conditions, the observed effects of **sentence complexity and sentence length** on pausing remain robust. Moreover, using a lower threshold may help detect subtle changes in timing and fluency that are salient in PD but could be overlooked with higher thresholds. Future work should empirically examine how pause threshold selection affects pausing metrics in PD and considers reporting multiple pause categories to facilitate comparability across studies.”

In lines 439-440, this sentence has several issues. First, it is not clear why pause duration would be affected by articulatory movements as the articulators are not active during silent intervals. Second, speech rate and articulation timing have been shown to be related to cognitive function in people with PD in several studies.

**Response:** Thank you for this comment. We have removed the referenced sentence and instead expanded the discussion in the manuscript to address the effects of respiratory and grammatical pauses, following your previous suggestion. These revisions can be found in lines 455-477 of the revised manuscript.

**Reviewer #2:** This is an interesting paper that has the potential to make a valuable contribution to the field. The introduction is very well written, but the results and discussion sections would benefit from some further detail and exploration of issues.

My detailed comments are as follows:

Line 157 – I'm not sure I agree with the classification of sentences into long and short. Was this also based on DeDe and Salis? I don’t think a sentence of 17 can be considered short, and one of 18 long just because the happen to lie above and below the mean. The authors should either present what the actual sentence length distribution was, showing that there was a sufficiently large gap between the two categories for the stats to make sense, or re-analyse the data with a different categorisation that results in a sufficient gap if that’s possible.

**Response:** We thank the reviewer for raising this important point and for prompting us to clarify our rationale. The classification of sentences into “short” and “long” was not intended to reflect an absolute or theory-driven notion of sentence length, but rather to operationalize relative length within the stimulus set used in this study. The split was based on the mean sentence length of the materials, which resulted in two groups that differed reliably in average length across the dataset.

We agree that, in absolute terms, a difference between sentences of 17 and 18 words is minimal, and we acknowledge that the labels “short” and “long” should therefore be interpreted as relative descriptors rather than categorical linguistic distinctions. Importantly, our statistical analyses do not rely on a presumed qualitative boundary between these categories, but on the systematic variation in sentence length across items. The use of a mean-based split is a common approach when sentence length is treated as a distributional property of a specific stimulus set rather than as a theoretically fixed category (Rucker et al., 2015).

To address this concern, we clarified this rationale in the manuscript and explicitly noted that the short vs. long distinction reflects a relative division of the materials rather than a claim about absolute sentence length classes. We also reported the full sentence-length distribution in the stimuli to make this transparent to the reader (see Figure 1). The distribution of sentences in the Rainbow passage:

- Short sentences (S): 11 sentences, ranging from 7-17 words (mean: 11.82 words)
- Long sentences (L): 8 sentences, ranging from 21-28 words (mean: 23.75 words)
- Gap between categories: 4 words (the shortest long sentence has 21 words, while the longest short sentence has 17 words)

The 4-word gap demonstrates that there is no overlap between the two categories, which is essential for valid statistical comparison. This clear separation means:

- Any differences found between groups can be confidently attributed to the sentence length manipulation
- There's no ambiguity about which category a sentence belongs to

However, we do not believe that a re-analysis with a different categorisation is warranted, as the current approach adequately captures length-related variation within the constraints of the existing materials and does not affect the interpretation of the results. Please refer to lines 169-180 in the Methds section in the manuscript and below:

“Sentence length was operationalised using a relative short vs. long distinction based on the distribution of the stimulus set (Rainbow passage), rather than on an absolute sentence length criterion. The distribution of sentences in the Rainbow passage:

- Short sentences (S): 11 sentences, ranging from 7-17 words (mean: 11.82 words)
- Long sentences (L): 8 sentences, ranging from 21-28 words (mean: 23.75 words)
- Gap between categories: 4 words (the shortest long sentence has 21 words, while the longest short sentence has 17 words)

The 4-word gap demonstrates that there is no overlap between the two categories, which is essential for valid statistical comparison. Figure 1 shows both the distribution of sentences across the passage and a clear range comparison demonstrating this separation.”

Figure 1.

Line 188 – not sure how a pause below 200 ms would reflect breath?

**Response:** Thank you for this comment. We realized that the original sentence was unclear, so we have rephrased it and also added a rationale for the choice of the 200 ms threshold, addressing Reviewer 1’s comment, as well. As we pointed in the manuscript pauses were set at a boundary of 200 ms, based on recommendations by DeDe and Salis (2020) to capture shorter pauses while excluding those that likely corresponded to breath or articulation. We have expanded here to include the rationale for pause duration threshold clear. Please see below and refer to lines 208-224:

“A TextGrid was created in Praat to mark silent pauses, defined as intervals exceeding 150 ms based on (Alvar et al., 2019). For this study, pauses were set at a boundary of 200 ms, based on recommendations by DeDe and Salis (2020), to capture shorter pauses while excluding those that likely corresponded to breath or articulation. A **200-ms pause duration threshold** is commonly justified in studies of PD speech because it provides a principled boundary between **brief articulatory gaps** and **functionally meaningful pauses** related to speech planning, timing, and cognitive–linguistic processing (Lee et al., 2019). Silent intervals shorter than approximately 200 ms are generally considered part of normal articulatory coordination and segmental timing, whereas pauses exceeding this duration are more likely to be perceived by listeners as disruptions to speech flow and to reflect higher-level processes such as lexical retrieval, syntactic planning, or respiratory control (Goldman-Eisler, 1968; Campione & Véronis, 2002). In PD, basal ganglia dysfunction is known to affect speech timing and sequencing, making pauses above this threshold particularly relevant as markers of impaired motor planning and cognitive–linguistic integration (Guenther, 2016; Ahn et al., 2014).Using a 200-ms cutoff therefore helps isolate pauses that are sensitive to PD-related speech impairments while excluding micro-silences that are unlikely to be clinically or theoretically meaningful, and this threshold has been widely adopted in analyses of connected speech in PD and related populations (Campione & Véronis, 2002; Lee et al., 2019).”

Line 216 – would it not have been safer to use non-parametric tests under these circumstances?

**Response:** We thank the reviewer for this suggestion. While the PD group did not meet normality assumptions, parametric repeated measures ANOVAs are generally considered **robust to violations of normality**, particularly when sample sizes are moderate to large (Pallant, 2020), as in our study. Additionally, our design included balanced group sizes and repeated measures, which further supports the robustness of the ANOVA. We therefore believe that the use of parametric tests is appropriate in this context. Nevertheless, we have clarified this rationale in the Methods section to make our choice explicit. Please refer to line 251-253 in the Methods section, and below:

“Although the PD group did not meet normality assumptions, parametric repeated measures ANOVAs were used because they are robust to violations of normality, particularly with moderate sample sizes and balanced repeated measures (Pallant, 2020).”

Line 225 - 227 – this sentence is misleading, I thought you were examining the relationship between cognition and dysarthria until I crosschecked with the research questions.

**Response:** Thanks for this comment. We have now rephrased this sentence to read: “Finally, within the PD group, Spearman’s correlations were conducted to examine the relationships between (1) dysarthria severity and the number and duration of pauses, and (2) cognitive ability (as measured by the MoCA) and the number and duration of pauses at different sentence levels.” Please refer to lines 261-264 in the Methods section.

Line 241 – would be useful to see the actual MLU mean and range here

**Response:** We thank the reviewer for this suggestion. Because the Rainbow Passage is a fixed reading stimulus, mean length of utterance (MLU) is constrained by sentence structure. As such, MLU primarily reflects production accuracy (e.g., omissions or truncations), and comparisons are therefore made relative to the expected sentence structure of the passage rather than treated as an independent outcome measure. For this reason, MLU was included as a descriptive control measure to characterize the task rather than as a primary result. We have now included descriptive information about the Rainbow Passage in the Study Design section, stating that “The Rainbow Passage consists of 19 sentences, with a mean length of utterance of 17.21 words (range: 8–35 words).” Please see lines 190-192.

Line 252 – you already said that in line 247?

**Response:** Thank you for pointing this out. We have removed this sentence in the revised manuscript.

Line 279 - slightly confused as you already reported group differences above, e.g. line 268- how is this different?

Line 301 – again these results seem to have already been reported above, see e.g. line 273. Are these tests done for each group? It’s confusing as in the RQ1 section you already refer to individual groups rather than the whole data. I suggest you just have one results section, presenting both ANOVA and any post-hoc tests done on individual group data and tease the results apart according to RQ in the discussion rather than repeating yourself.
Or make it clearer what’s new in the sections that follow the initial presentation and remove any doubled up results from the relevant part.
Overall it’s quite difficult to follow all the descriptive data and stats results, could you present them in a summary table?

**Response:** Thank you for this observation. We have restructured the Results section to consolidate the main ANOVA and post-hoc analyses into a single section and have added a summary table (Table 2) to improve readability. Please refer to lines 266–334 of the revised Results section.

Line 331 – highlight again in the text that this refers only to the PD group as it’s easily missed if you don’t read the long heading

**Response:** Thanks for pointing this out. This now has been added to the revised version of the manuscript. See line 336.

Line 403 – so did you have any evidence of this happening in individuals? Pauses are generally excessively long if there is an initiation problem, rather than a few hundred milliseconds.

**Response:** In the present study, we did not examine individual differences in speech initiation difficulties in relation to pause behaviour in PD. Future studies could explicitly address this issue to disentangle initiation-related deficits from pause characteristics and to better understand how initiation difficulties may contribute to pausing in PD. We have added this point to the Discussion section (see lines 410-414 of the revised manuscript), as outlined below.:

“Nonetheless, in the present study, we did not examine individual differences in speech initiation difficulties in relation to pause behaviour in PD. Future studies could explicitly address this issue to disentangle initiation-related deficits from pause characteristics and to better understand how initiation difficulties may contribute to pausing in PD.”

Line 405 – so were all simple sentences short then?

**Response:** Based on the definitions of sentence complexity (simple vs. complex) and sentence length (short vs. long), most simple sentences were also short; however, there was one exception. Specifically, the sentence “These take the shape of a long, round arch, with its path high above and its two ends apparently beyond the horizon” was classified as simple despite being long in terms of word count.

Line 407 – fewer pauses in which context/sentence type? Or in general?

**Response:** Here we meant in general. Based on your next comment, we have now revised this section. Please see below.

Line 408 – you might want to consider here the effects of uncontrolled speeding up and decreasing range of movement, i.e. PwPD have problems pacing themselves and stopping which could potentially cause this effect

**Response:** This is a valuable point to this section of the Discussion section. We have now revised this section to read (see lines 415-441 in the revised manuscript):

**“**Interestingly, no significant differences were found in the duration of pauses in short, simple, and complex sentences, nor in the mean number of pauses in simple sentences in PD compared to the OC group. Although many studies report increased pause frequency or duration in PD, others have shown reduced or fewer pauses, especially in contexts where speech rate accelerates or rhythmic timing is disrupted (Tajden & Wilding, 2011; Skodda & Schlegel, 2008; D’Ascanio et al., 2025). For example, PD speakers exhibited **fewer overall pauses and longer silent pauses**, with a reduction in filled pauses compared to controls, suggesting impaired motor planning and automatic responses in pausing behaviour in PD (e.g., Hammen & Yorkston, 1996). Similarly, accelerated speech in PD has been associated with **reduced total pause time and fewer within‑word pauses,** indicating that dysregulated pacing and timing can lead to compressed pause patterns (e.g., Skodda & Schlegel, 2008). Although the reason for this discrepancy with our findings remains unclear, it could be that uncontrolled speeding of speech (speech festination) and difficulty terminating ongoing motor actions may lead to **compressed speech with fewer or absent planned pauses**, particularly at linguistically appropriate boundaries (Ackermann & Ziegler, 1991; Skodda & Schlegel, 2008). In such cases, similar or reduced pausing in PD compared to controls may reflect impaired motor pacing rather than increased fluency. Conversely, reduced movement range, impaired respiratory–speech coordination, and increased planning demands may result in **more frequent or longer compensatory pauses**, especially in longer or more complex utterances (Solomon & Hixon, 1993; Duffy, 2013). The effects of sentence complexity and sentence length on the number and duration of pauses were consistent with predictions, with both factors contributing significantly to the differences observed between PD and control speakers. As expected, increased sentence complexity was associated with more pauses, likely due to the greater cognitive load required to process more complex structures. For PD speakers, this cognitive demand likely results in the production of smaller linguistic units, leading to more frequent pauses, compounded by impaired physiological control of respiration and motor control (Huber et al., 2012). Together, these findings suggest that pausing in PD reflects a dysregulation of speech timing and motor control, with observed patterns depending on task demands, sentence characteristics, and individual differences in motor impairment.”

Line 410 – stats between PD and controls or different sentence types?

**Response:** We have now revised this section and omitted this sentence.

Line 415 could you separate your discussion of between sentence and between group effects?

**Response:** Thanks for this comment. We have restructured this paragraph and integrated it with the preceding paragraph to include the main discussions of between group effect and separate it from the between sentence effect. Please refer to lines 432-441.

Line 419 – so some research suggests more frequent pauses then, contrasting with Line 407? This needs further explanation if this is based on the speaking task etc.

**Response:** We have restructured this paragraph and integrated it with the preceding paragraph to highlight the key points that the literature reports contrasting findings regarding both the number and duration of pauses in individuals with PD compared to control participants. Please refer to lines 432-441.

Line 422 – it’s not really a contradiction, your controls just didn’t have cognitive decline, so you wouldn’t expect that effect.

**Response:** We agree with the reviewer’s comment. We have now revised this section and excluded that this is a contradiction.

Line 426 – the paragraph would benefit from a definition of constituent length vs information load, you seem to be mixing the two up further down in line 430, or at least assume that they are related to each other.

**Response:** Thank you for this comment. We agree that constituent length and information load should be clearly distinguished. We have therefore added an explicit definition of both terms earlier in the paragraph. By constituent length we mean the linear size of a constituent (measured in number of words), whereas information load refers to the amount of information or processing demand associated with a constituent. While these notions are often correlated, they are not equivalent. We have revised line 430 to clarify that our argument relies on their relationship rather than treating them as interchangeable. Please refer to lines 443-446 and lines 447-451 and see below:

“Constituent length is the linear size of a constituent, measured as the number of words it contains, whereas information load refers to the amount of information or processing demand associated with a constituent. Although these notions are often correlated, they are conceptually distinct and not equivalent.”

“However, this study did not control for constituent length, measured as syllable count, making it unclear whether the observed effects reflect information load or length-based processing factors. Although these variables are often correlated, they are conceptually distinct, and future work will be necessary to disentangle their individual contributions.”

Line 437 – why? This needs more detail. This whole paragraph, combined with the next one needs, further exploration – your work has the potential to contribute to the discussion of whether pausing behaviour reflects motor speech difficulties or sentence processing, i.e. cognitive issues, but this is not really explored here. I suggest you look at some of the literature focusing on language difficulties in PwPD and what they found re pausing behaviour to go into more detail here.

**Response:** Thank you for this comment, which was also raised by Reviewer 1. We have revised and expanded this paragraph to clarify the relevant issues, with a greater focus on motor speech difficulties as well as language and grammatical factors. Please see lines 455–477 and the text below.

“Previous research has shown that individuals with PD exhibit longer and more frequent silent pauses, reduced use of filled pauses, and poorer alignment of respiratory pauses with grammatical boundaries, reflecting disruptions in linguistic planning, automatic cueing, and respiratory–speech coordination (Rosen et al., 2010; Huber & Darling-White, 2017; Lowit et al., 2018). Much of this evidence comes from spontaneous or semi-spontaneous speech tasks, such as narratives or picture descriptions, which place high demands on lexical retrieval, discourse planning, and internal timing, thereby amplifying pause abnormalities in PD (Rosen et al., 2010; Ash et al., 2012). Silent pauses in PD have been associated with increased cognitive–linguistic load and lexical access difficulty, whereas filled pauses—typically serving as planning buffers—are reduced, likely due to basal ganglia dysfunction affecting internally generated cues (Rosen et al., 2010; Ash et al., 2012). This study extends this literature by examining pausing during a controlled reading task (Rainbow Passage), in which lexical content and syntactic structure are externally provided, reducing discourse-level planning demands while preserving sentence-level formulation and respiratory coordination requirements. Despite these reduced demands, sentence complexity (simple vs. complex) and sentence length (short vs. long) systematically modulated pause behavior in PD, with longer and more complex sentences eliciting longer silent pauses, particularly within sentences. Although respiratory pauses were not directly measured, the altered distribution of silent pauses within grammatical structures is consistent with prior findings of impaired coordination between respiration and linguistic planning in PD (Lowit et al., 2018). Together, these findings indicate that pause abnormalities in PD are evident across speech task types and reflect an interaction between motor–respiratory constraints and higher-level linguistic planning, with sentence complexity and length exacerbating these effects even during passage reading.”

Line 445 – did Hammen and Yorkston only refer to speech initiation? There are plenty of other reasons this could be happening

**Response:** Thank you for this comment. Hammen and Yorkston (1996) discussed delayed speech initiation as one possible explanation for increased pause duration in speakers with more severe dysarthria; however, we agree that this is unlikely to be the only contributing factor. Increased pause duration may also arise from reduced speech rate, increased articulatory and linguistic planning demands, respiratory–phonatory constraints, or more general motor control limitations associated with dysarthria severity. We have revised the manuscript to clarify that delayed speech initiation is only one possible mechanism and to acknowledge these additional explanations. Please refer to the lines 478-487 and see below:

“Dysarthria severity was positively correlated with the duration of pauses in short, long, and complex sentences, with borderline significance in simple sentences. This finding is consistent with previous work by Hammen and Yorkston (1996), who reported longer pause durations in speakers with more severe dysarthria and suggested delayed speech initiation as one possible contributing factor. However, increased pause duration may also reflect other mechanisms, including reduced speech rate, increased articulatory or linguistic planning demands, respiratory–phonatory constraints, or more general motor control limitations. Further investigation is therefore warranted, particularly with respect to the relationship between dysarthria severity, pause behavior, and speech rate, which was not controlled for in the present study.”

Line 446 - don’t follow this statement, where does speech rate suddenly come from? And why wasn’t that measured if it’s important, doesn’t take that long to extract.

**Response:** Thank you. The statement has been revised in accordance with your previous comment (see above).

Line 451 – how to you expect to blind the experimenter to group membership when they can hear that the person has a speech disorder?

**Response:** Blinding the experimenter would be relevant if not all participants with PD had dysarthria. We did not exclude participants with PD who lacked dysarthria; in practice, however, group membership was generally apparent.

Line 453 – adaptation would only be an issue if the participant sat and practised reading the passage several times before you recording – how likely was that? Normally you would ask a person to read through a passage silently before reading aloud to avoid any reading issues affecting speech performance anyway, so I don’t think this is a big issue.

**Response:** Thank you for this comment. As the materials were provided to participants in advance, we cannot be certain whether they practiced the passage prior to the recording. Therefore, we cannot rule out the possibility that prior familiarity may have influenced their performance.

Line 458 – don’t understand what the issue is here, what are speech initiation measurements? And did you use a script without checking for accuracy afterwards?

**Response:** Thank you for raising this point. By “speech initiation measurements,” we are referring to the time between the prompt to begin speaking and the actual onset of speech. While Praat was used to analyze pauses, adjustments to silence boundaries could have slightly affected the precision of both these onset times and pause durations. All recordings followed a standardized script, and although we did not formally check each reading for strict accuracy, inter-rater reliability was excellent, indicating that the measurements were consistent across raters despite these minor limitations. We have now added to this to the Limitation and future directions section, see lines 513-517, and see below:

“While Praat was used to analyze pauses, adjustments to silence boundaries could have slightly affected the precision of both these onset times and pause durations. All recordings followed a standardized script, and although we did not formally check each reading for strict accuracy, inter-rater reliability was excellent, indicating that the measurements were consistent across raters despite these minor limitations.”

Line 461 – if this is an issue I suggest you go over your stats results again that were not significant to see whether there was any potential for differences without the correction and discuss this in the text.

**Response:** We appreciate this suggestion. Upon consideration, we determined that this point does not substantially affect the interpretation of our findings and have therefore removed it from the Limitations and future directions section.

Line 464 – You need to specify which one of your observations this is based on

**Response:** Thank you for this helpful suggestion. To ensure that all statements are fully supported by our data, we have removed this point from the Limitation and future directions section.

Line 467 – this seems a bit far fetched unless you have some references supporting this or provide some more detail for why you think this might be the case?

**Response:** Thank you for this comment. We agree that suggestions for future work should be supported by evidence. Recent research has identified distinct speech subtypes in Parkinson’s disease—such as prosodic, phonatory‑prosodic, and articulatory‑prosodic patterns—using quantitative acoustic analyses, indicating that different aspects of speech production (including timing and prosody) may vary systematically across subgroups of dysarthria in PD (Rusz et al., 2021). Given these distinct profiles, examining whether features such as silent pause characteristics are consistent or differ across subtypes could plausibly contribute to more refined diagnostic screening tools. We have now expanded on this sentence, see lines 545-548, and below:

“Additionally, research could examine whether silent pauses are consistent across different speech subtypes of Parkinson’s disease—such as prosodic, phonatory‑prosodic, and articulatory‑prosodic patterns—potentially contributing to the development of diagnostic screening tools (Rusz et al., 2021).”

Line 470 – how do you separate cognitive demands and sentence complexity?

**Response:** We appreciate the reviewer’s insightful comment. In the revised manuscript, we have removed references to cognitive demands, as they were not analyzed separately in this study. The findings now focus solely on sentence complexity and length and their influence on the production of silent pauses in PD speech. This revision clarifies the scope of our analysis and addresses the concern raised.

Line 474 – are you actually suggesting that silent pause measures would be more valid to prioritise patients than e.g. their intelligibility, impact of their dysarthria etc.?

**Response:** We thank the reviewer for this comment. We would like to clarify that we do **not** suggest that silent pause measures are more valid than established clinical measures such as intelligibility or functional impact of dysarthria. The sentence in the original manuscript was intended to indicate that further research is needed to explore whether silent pauses could have **complementary utility** in prioritizing patients or assessing treatment outcomes, alongside existing measures. We have revised the manuscript to make this point clearer. Please refer to the lines 553-555, and see below:

“Further research is needed to explore whether silent pauses could provide complementary information to existing clinical measures, such as intelligibility and the functional impact of dysarthria, in prioritizing patients or assessing treatment outcomes.”

Line 476 – and what would that look like in terms of actual treatment provided? I would avoid such generic statements unless they have actually been explored in the text a bit more.

**Response:** We thank the reviewer for this comment. We agree that the statement was too general and not directly supported by the data presented. We have therefore removed the sentence suggesting that understanding the theoretical underpinnings may lead to better therapeutic strategies.

References:

1. Ackermann, H., & Ziegler, W. (1991). Articulatory deficits in Parkinsonian dysarthria: An acoustic analysis. Journal of Neurology, Neurosurgery & Psychiatry, 54(12), 1093–1098. <https://doi.org/10.1136/jnnp.54.12.1093>
2. Ahn, J. S., Sidtis, D. V. L., & Sidtis, J. J. (2014). Effects of deep brain stimulation on pausing during spontaneous speech in Parkinson’s disease. Journal of Medical Speech-Language Pathology, 21(3), 179.
3. Alvar, A. M., Lee, J., & Huber, J. E. (2019). Filled pauses as a special case of automatic speech behaviors and the effect of Parkinson's disease. American Journal of Speech-Language Pathology, 28(2S), 835–843.
4. Andrade, E. I. N., Manxhari, C., & Smith, K. M. (2023). Pausing before verb production is associated with mild cognitive impairment in Parkinson’s disease. Frontiers in Human Neuroscience, 17, 1102024.
5. Ash, S., McMillan, C., Gross, R. G., Cook, P., Gunawardena, D., Morgan, B., … Grossman, M. (2012). Impairments of speech fluency in Lewy body spectrum disorder. Brain and Language, 120(3), 290–302.
6. Blanchet, P. G., & Snyder, G. J. (2009). Speech rate deficits in individuals with Parkinson’s disease: A review of the literature. Journal of Medical Speech-Language Pathology, 17(1), 1–7.
7. Bohsali, A., & Crosson, B. (2016). The basal ganglia and language: A tale of two loops. In J.-J. Soghomonian (Ed.), The basal ganglia: Novel perspectives on motor and cognitive functions (pp. 217–242). Springer. <https://doi.org/10.1007/978-3-319-42743-0_10>
8. Campione, E., & Véronis, J. (2002). A large-scale multilingual study of silent pause duration. Speech Communication, 36(1–2), 63–82. <https://doi.org/10.1016/S0167-6393(01)00034-0>
9. D’Ascanio, S., Piras, F., Spada, C., Pellicano, C., & Piras, F. (2025). Pauses as a quantitative measure of linguistic planning challenges in Parkinson’s disease. Brain Sciences, 15(11), 1131.
10. DeDe, G., Salis, C., & Bose, A. (2025). Discourse planning and production in post-stroke aphasia: The role of executive function on pauses and clauses. Aphasiology, 1–24.
11. Fumel, J., Bahuaud, D., Weed, E., Fusaroli, R., & Basirat, A. (2024). A systematic review and Bayesian meta-analysis of acoustic measures of prosody in Parkinson's disease. Journal of Speech, Language, and Hearing Research, 67(8), 2548–2564.
12. Goetz, C. G. (2009). Movement disorder society–unified Parkinson’s disease rating scale (MDS-UPDRS): A new scale for the evaluation of Parkinson’s disease. Revue Neurologique, 166(1), 1–4.
13. Goldman-Eisler, F. (1968). Psycholinguistics: Experiments in spontaneous speech. Academic Press.
14. Guenther, F. H. (2016). Neural control of speech. MIT Press. <https://doi.org/10.7551/mitpress/10471.001.0001>
15. Hammen, V. L., & Yorkston, K. M. (1996). Speech and pause characteristics following speech rate reduction in hypokinetic dysarthria. Journal of Communication Disorders, 29(6), 429–445.
16. Hoehn, M. M., & Yahr, M. D. (1967). Parkinsonism: Onset, progression, and mortality. Neurology, 17(5), 427–442. <https://doi.org/10.1212/WNL.17.5.427>
17. Huber, J. E., & Darling-White, M. (2017). Changes in respiratory patterns during speech in individuals with Parkinson’s disease. Journal of Speech, Language, and Hearing Research, 60(2), 360–373. <https://doi.org/10.1044/2016_JSLHR-S-15-0389>
18. Illner, V., Tykalová, T., Novotný, M., Klempíř, J., Dušek, P., & Rusz, J. (2022). Toward automated articulation rate analysis via connected speech in dysarthrias. Journal of Speech, Language, and Hearing Research, 65(4), 1386–1401.
19. Knowles, T., Adams, S. G., & Jog, M. (2021). Variation in speech intelligibility ratings as a function of speech rate modification in Parkinson's disease. Journal of Speech, Language, and Hearing Research, 64(6), 1773–1793.
20. Lee, J., Huber, J., Jenkins, J., & Fredrick, J. (2019). Language planning and pauses in story retell: Evidence from aging and Parkinson’s disease. Journal of Communication Disorders, 79, 1–10.
21. Lowit, A., Marchetti, A., Corson, S., & Kuschmann, A. (2018). Rhythmic performance in hypokinetic dysarthria: Relationship between reading, spontaneous speech and diadochokinetic tasks. Journal of Communication Disorders, 72, 26–39.
22. Miller, I. N., & Cronin-Golomb, A. (2010). Gender differences in Parkinson's disease: Clinical characteristics and cognition. Movement Disorders, 25(16), 2695–2703.
23. Moreau, C., & Pinto, S. (2019). Misconceptions about speech impairment in Parkinson's disease. Movement Disorders, 34(10), 1471–1475.
24. Rosen, K., Murdoch, B., Folker, J., Vogel, A., Cahill, L., Delatycki, M., & Corben, L. (2010). Automatic method of pause measurement for normal and dysarthric speech. Clinical Linguistics & Phonetics, 24(2), 141–154.
25. Rucker, D. D., McShane, B. B., & Preacher, K. J. (2015). A researcher's guide to regression, discretization, and median splits of continuous variables. Journal of Consumer Psychology, 25(4), 666–678.
26. Rusz, J., Tykalova, T., Novotny, M., Zogala, D., Sonka, K., Ruzicka, E., & Dušek, P. (2021). Defining speech subtypes in de novo Parkinson disease: Response to long-term levodopa therapy. Neurology, 97(21), e2124–e2135.
27. Skodda, S., & Schlegel, U. (2008). Speech rate and rhythm in Parkinson’s disease. Movement Disorders, 23(7), 985–992. <https://doi.org/10.1002/mds.21996>
28. Solomon, N. P., & Hixon, T. J. (1993). Speech breathing in Parkinson’s disease. Journal of Speech, Language, and Hearing Research, 36(2), 294–310.
29. Tjaden, K., & Wilding, G. (2011). Speech and pause characteristics associated with voluntary rate reduction in Parkinson's disease and multiple sclerosis. Journal of Communication Disorders, 44(6), 655–665.
30. Van Den Eeden, S. K., Tanner, C. M., Bernstein, A. L., Fross, R. D., Leimpeter, A., Bloch, D. A., & Nelson, L. M. (2003). Incidence of Parkinson’s disease: Variation by age, gender, and race/ethnicity. American Journal of Epidemiology, 157(11), 1015–1022.
31. Whitfield, J. A., & Gravelin, A. C. (2019). Characterizing the distribution of silent intervals in the connected speech of individuals with Parkinson disease. Journal of Communication Disorders, 78, 18–32.
