# Supplementary material for: Pause characteristics of sentence production in Parkinson’s disease: Insights from sentence complexity and length
Source: PLoS One. 2026 Apr 23;21(4):e0330808. doi: 10.1371/journal.pone.0330808 (PMC13105342; doi:10.1371/journal.pone.0330808)
Supplement: Attachment — Submitted filename: Response to Reviewers_R2.docx [file pone.0330808.s009.docx]

**Manuscript ID:** PONE-D-25-42565R1

Responses to the reviewers’ comments

We thank the reviewers and Editors of *PLOS ONE* for their continued time and constructive feedback. In response to the second round of review, we have carefully considered each point and made further revisions to improve clarity and completeness, which are highlighted in red in the revised manuscript. Detailed point-by-point responses are provided below, and we remain committed to addressing any additional concerns to strengthen the manuscript.

**Reviewer #1:** The authors have done very well in their revisions, and have thoroughly and thoughtfully addressed most of my comments. The manuscript is substantially improved. Additional points I am hoping the authors can clarify in the discussion are:

1. In Lines 399-404, I do not understand how references 41 and 55 are being interpreted in the context of the findings just presented. Are the authors saying that both papers support that both cognitive and motor function are associated with fluency and pausing, or is the intended meaning something more specific? In particular, the last line at 403-404 is confusing. I do not know what "factors" need to be investigated further.

**Response:** Thanks for this thoughtful comment, we understand that these sentences may be confusing. We have now re-phrased them to read as below. Please see lines 391-398 in the revised manuscript:

“It has been found that pause duration correlates with **executive functioning, grammatical ability, and motor initiation,** indicating that **linguistic planning and executive control — not just motor capacity — influence how long speakers pause [23].** Krivokapić [43] also suggests that sentence length may influence both pre- and post-boundary pause durations, which increases processing effort and planning time. This could explain the observed increase in between-sentence pauses. However, future studies need to investigate linguistic, motor, and cognitive factors to better understand their relative contributions to pause durations.”

2. The section on speech acceleration (Lines 420-430) would benefit from some additional clarification. My impression was that in this study, PD participants globally had an increased number and duration of pauses compared to older controls, so I am not sure what data supports festination or acceleration of speech timing in this present work.

**Response:** Thank you for this comment. We have now clarified this point to mention that those previous findings on festination and acceleration of speech timing are at odds with our findings and provided a possible explanation for it with more clarity. See blow and lines 419-424 in the revised manuscript:

**“**These findings are inconsistent with ours. The reason for this discrepancy remains unclear. However, one possible explanation is that uncontrolled acceleration of speech (speech festination) and difficulty terminating ongoing motor actions—both of which may occasionally occur in individuals with PD speech—could lead to compressed speech characterized by fewer, or even absent, planned pauses, particularly at linguistically appropriate boundaries [22,59]. However, we did not observe this pattern in our own data.”

3. Looking more closely at the single/complex sentence findings, I would consider adding to your discussion the possibility that the rainbow passage was not the optimal experimental paradigm to assess grammatical complexity and pausing, thus further research is needed. I understand the approach used to classify the sentences, but it is difficult to know how well balanced these sentences may be from other articulatory and linguistic perspectives, and also there is not an even number of simple vs. complex.

**Response:** Thank you for bringing up this point. We have added a paragraph in the **Limitation and future directions** section that addresses this comment. Please see blew and lines 51--529 in the revised manuscript:

**“**The Rainbow Passage may not be the optimal paradigm for isolating the effects of grammatical complexity and sentence length on pausing. Although we classified sentences according to established criteria, the passage was not designed to systematically control syntactic structure or to orthogonally manipulate sentence length and complexity. As a result, most simple sentences were short, with only one notable exception. We chose to examine both variables because they capture related but distinct aspects of speech production: **sentence length reflects planning demands associated with producing longer sequences of words or syllables**, whereas **sentence complexity reflects hierarchical syntactic processing and higher-level linguistic planning**. The overlap between these variables means that some observed effects on pause number and duration may not be fully independent. Nevertheless, analyzing both provides complementary insight into how different linguistic and production constraints contribute to pausing in PD. Sensitivity analyses indicate that the overall patterns—more frequent and longer pauses in PD—are robust, but we acknowledge that the overlap limits the ability to fully disentangle the separate contributions of length and complexity. In addition, the sentences may not be fully balanced with respect to other articulatory, prosodic, or lexical factors, and the unequal number of simple versus complex sentences further constrains direct comparisons. Future studies using experimentally controlled stimuli that systematically vary sentence length and complexity independently would allow for a more precise evaluation of their individual and interacting effects on pausing.”

**Reviewer #2:** PONE comments
Thank you to the authors for their careful consideration of the reviewer comments, generally I am happy that the issues have been addressed and I only have some minor comments on the revised material as outlined below. However, one of the responses re. sentence length/complexity has opened up a new area of debate with regard to research question 2 which requires more detailed consideration, again see comment below.

L73-75 – two sentences don’t work together – one is on management, the other on speech characteristics

**Response:** Thanks for this comment. We have revised the second sentence. Please see lines 74-77 in the revised version of the manuscript, and below:

“However, these approaches primarily focus on global speech outcomes, and a more detailed understanding of how PD affects specific components of speech—such as pause behaviour—is still limited. This gap may constrain the development of more targeted and mechanism-based intervention strategies.”

L 85-88 I appreciate there are reference to support this statement, however, the description here is too one-sided , i.e. 1, the authors should also mention the fact that PwPD might have accelerated rate, and 2, there are definitely articulatory problems, such as undershoot, maybe they just don’t feature as much in milder patients.

**Response:** We appreciate this comment, and have revised the manuscript to acknowledge the accelerated rate and articulatorily problems in PD. Pease see below and refer to the lines 87-92 in the revised manuscript:

**“**Speech rate in PD is frequently reported as reduced, although findings are mixed and influenced by task type and disease stage; in some cases, individuals with PD may also exhibit an accelerated or festinating speech rate [19-21]. While articulation rate is often described as relatively preserved, particularly in milder stages, articulatory impairments such as reduced movement amplitude and articulatory undershoot may also be present [22].”

L88-91 more evidence needed that pausing behaviour is more robust beyond a DBS study

**Response:** Thank you for this comment. We have added more references to support this statement. Please see below and lines 92-98.

“Increased frequency and duration of pauses have been observed across a range of speech tasks, including spontaneous speech and reading, and have been associated with reduced speech fluency and executive-linguistic demands [e.g., 19–21, and 23–26]. These findings are further supported by evidence that modulation of basal ganglia–cortical circuits through deep brain stimulation can influence pause characteristics [27, 28:280–283], suggesting that pausing behaviour may reflect disruption of neural systems involved in speech sequencing and timing.”

L91-94 some repetition here and in the next section

**Response:** Thank you for this observation. We have revised this section and removed this sentence. Please refer to lines 99-106 in the revised version of the manuscript, and see below:

“Together, these findings suggest that both higher-level timing processes and segmental articulatory control can be affected in PD, with their relative contributions varying by individual and disease severity. Altered pause duration and distribution are consistently reported and may represent a clinically relevant feature of PD speech. These observations highlight the importance of moving beyond global speech rate measures toward more detailed analyses of pause timing in connected speech, and suggest that interventions targeting planning, prosodic phrasing, and respiratory–speech coordination may be beneficial [29, 30; see also 31].”

L150 sorry if I missed this but what was the dysarthria severity rating based on?

**Response:** Thank you for pointing this out. The dysarthria severity rating was already included in the original manuscript and was based on the 7-point scale by Duffy (2005). Please refer to the **Procedure section of the Materials and Methods (lines 243-246)** for the full details and below:

“Passage recordings were additionally analyzed for clinical dysarthria severity, rated by a trained Speech and Language Therapist (SLT) using a 7-point scale (1 = normal speech, 7 = severe speech) based on perceptual characteristics related to articulation, resonance, prosody, phonation, and respiration [54].”

L265 the results section is really difficult to read with all the stats results in the text. They seem redundant given that a summary is provided in Table 1, so I would suggest removing them from the text. I also wonder whether it would be more logical to report the post-hoc results in the same paragraph as the ANOVA so that they are grouped by variable rather than statistical analysis method, but I leave that up to the authors to decide.

**Response:** We thank the reviewer for this helpful suggestion. We have revised the **Results** section to improve clarity and readability. Specifically, we reduced the amount of detailed statistical reporting in the main text, retaining only the direction and significance of effects while referring readers to Table 2 for full statistical details. In addition, we reorganized the section to group ANOVA and post-hoc findings by outcome variable (pause number and pause duration) rather than by statistical procedure. We believe this restructuring substantially improves the logical flow and accessibility of the **Results** section while preserving transparency of reporting. Please see lines 276-321 in the revised version of the manuscript.

Previous L405 (see response to reviewer 2): I do not agree that the long sentence you refer to here is a simple sentence. Whilst there is no clear subordinate clause with a new verb here, the prepositional phrase starting with “with…” is not the simple phrase you normally encounter, such as in “I saw a man with a dog”. In fact, you could reinsert a verb “with it’s path being high above” which clearly denotes it as a subordinate clause. Whether you agree with this analysis or not, the sentence definitely stands out from the rest of your simple sentences and I would therefore check whether the results somehow bias the rest of the set and potentially exclude it from analysis as an anomaly.

**Response:** We thank the reviewer for this careful reading. We agree that the sentence *“These take the shape of a long, round arch, with its path high above and its two ends apparently beyond the horizon”* is atypical among the simple sentences and contains a complex prepositional phrase that could be interpreted as a reduced subordinate clause. However, according to our predefined criteria for simple versus complex sentences—where a simple sentence contains a single independent clause and may include compound nouns or verbs, and a complex sentence requires at least one dependent clause introduced by a subordinating conjunction or relative pronoun—this sentence meets the criteria for a simple sentence, as it has only one independent clause and no full subordinate clause with an explicit verb.

Importantly, to ensure that this atypical sentence does not bias the results, we conducted a sensitivity analysis excluding it. Re-running the ANOVAs for pause number and duration with this sentence removed did not materially change the pattern or significance of the results (all main effects and interactions reported in Table 2 remained significant). Therefore, while we acknowledge that the sentence is somewhat atypical, it does not appear to influence the overall findings and was retained in the analyses for completeness and consistency with the standardized Rainbow Passage.

Next, having established that with the exception of that one sentence, your simple sentences all fall into the short category and complex one in the long category, what exactly is the point of analysing both of these variables? If you wanted to differentiate between more/longer pauses being caused by higher level linguistic processing constraints versus speech production limitations, then this should have been controlled better by also including short complex sentences, or long, simple ones (e.g. item lists) which is obviously difficult to do in a standard reading passage. I would therefore like to see a much clearer argument for why both variables were considered and an exploration of the extent to which the results were influenced by the overlap in the discussion if it is decided that they should both remain.

**Response:** We thank the reviewer for this important point. We agree that, in the Rainbow Passage, there is substantial overlap between sentence length and sentence complexity, with most simple sentences being short and most complex sentences being long. Our rationale for analyzing both variables was to capture complementary aspects of speech production: sentence length reflects planning load related to the number of words or syllables, whereas sentence complexity reflects syntactic or hierarchical linguistic processing demands. While the standard Rainbow Passage does not allow for fully crossing length and complexity (e.g., short complex or long simple sentences), examining both variables provides descriptive insight into how these different linguistic and production factors may influence pausing.

To address the reviewer’s concern about overlap, we explored the results with respect to the relationship between length and complexity. The patterns of increased pause number and duration were broadly consistent across analyses, suggesting that both factors contribute to pausing, but we acknowledge that we cannot fully disentangle the effects of length versus syntactic complexity in this dataset. We have added a discussion of this limitation and its implications for interpreting our findings, highlighting that future studies could benefit from experimental designs that orthogonally manipulate sentence length and complexity to better isolate their contributions to pausing. Please see lines 510-529 in the **Limitations and future directions** section, and see below:

**“**The Rainbow Passage may not be the optimal paradigm for isolating the effects of grammatical complexity and sentence length on pausing. Although we classified sentences according to established criteria, the passage was not designed to systematically control syntactic structure or to orthogonally manipulate sentence length and complexity. As a result, most simple sentences were short, with only one notable exception. We chose to examine both variables because they capture related but distinct aspects of speech production: **sentence length reflects planning demands associated with producing longer sequences of words or syllables**, whereas **sentence complexity reflects hierarchical syntactic processing and higher-level linguistic planning**. The overlap between these variables means that some observed effects on pause number and duration may not be fully independent. Nevertheless, analyzing both provides complementary insight into how different linguistic and production constraints contribute to pausing in PD. Sensitivity analyses indicate that the overall patterns—more frequent and longer pauses in PD—are robust, but we acknowledge that the overlap limits the ability to fully disentangle the separate contributions of length and complexity. In addition, the sentences may not be fully balanced with respect to other articulatory, prosodic, or lexical factors, and the unequal number of simple versus complex sentences further constrains direct comparisons. Future studies using experimentally controlled stimuli that systematically vary sentence length and complexity independently would allow for a more precise evaluation of their individual and interacting effects on pausing.”

L516 – doesn’t make sense to me, two different raters putting the same speech samples through the same script should not show any differences, Praat does not change its analysis just because another rater presses the run button. What exactly was being examined here?

**Response:** We thank the reviewer for pointing this out. To clarify, while Praat automatically detects silences, we visually inspected each recording and **manually adjusted silence boundaries** as needed to ensure accurate identification of pause onsets and offsets. Inter-rater reliability was calculated on these manual adjustments, and the excellent agreement indicates that both raters made highly consistent judgments despite the need for occasional visual correction. We have made this clearer in the manuscript. We moved this to the **Procedure** section of **Materials and methods**. Please see lines 247-253 and below:

“While Praat was used to initially identify pauses, each recording was visually inspected by two independent raters, and silence boundaries were manually adjusted as needed to ensure accurate determination of pause onsets and offsets. Inter-rater reliability for these adjustments was assessed using qualitative ratings for both the number and duration of pauses, yielding excellent agreement (reliability coefficients: 0.93 for pause number and 0.86 for pause duration) [55], indicating that measurements were consistent across raters despite the minor manual corrections.”

L527 above you have included a correlational analysis of dysarthria severity with MoCA scores, so clearly you did more than set a score range as an inclusion criterion. Also, which particular cognitive features would you suggest to investigate to differentiate speech from cognitive effects? I think you might also need to rethink your speech task if this an aim.

**Response:** We thank the reviewer for this comment and apologize for any lack of clarity. To clarify, in our study we conducted separate correlational analyses between MoCA scores and pause characteristics, and between dysarthria severity and pause characteristics; we did not analyze a direct correlation between MoCA and dysarthria severity. While restricting MoCA scores limited gross cognitive impairment, more detailed cognitive assessment—including attention, working memory, executive function, and language processing—would be informative to better dissociate motor and cognitive contributions to pausing. We also agree that task design is important in this context, and—as noted in our response to the earlier comment regarding the Rainbow Passage—the current reading task limits the ability to fully separate linguistic, cognitive, and motor demands. Future work using more targeted and experimentally controlled speech tasks would allow clearer differentiation of these factors. Please see lines 539-547 in the revised manuscript and below:

“Although cognitive status was partially controlled by restricting the range of MoCA scores in the PD group, comprehensive neuropsychological assessments were not conducted. In the current study, correlations were examined between MoCA scores and pause characteristics, as well as between dysarthria severity and pauses, allowing for initial exploration of cognitive and motor contributions. However, more detailed assessment of specific cognitive domains—such as attention, working memory, executive function, and language processing—would help to better dissociate speech motor impairments from cognitive-linguistic influences on pausing. Future work may also benefit from designing speech tasks that more precisely isolate motor versus cognitive contributions to speech timing.”

L553 be specific, only pause duration showed any link to dysarthria severity

**Response:** Thanks for this comment. We revised this sentence to be more specific and point to pause duration. Please see lines 567-571 in the revised manuscript and below:

“Clinically, these findings indicate that silent pause duration could serve as an objective measure of dysarthria severity and treatment outcomes. Further research is needed to explore whether silent pause duration could provide complementary information to existing clinical measures, such as intelligibility and the functional impact of dysarthria, in prioritizing patients or assessing treatment outcomes.”
